# Docking experiments suggest that gloriosine has microtubule-targeting properties similar to colchicine

**DOI:** 10.1038/s41598-023-31187-6

**Published:** 2023-03-24

**Authors:** Ankita Misra, Mridul Kant Chaudhary, Satyendra Pratap Singh, Deepali Tripathi, Saroj Kanta Barik, Sharad Srivastava

**Affiliations:** 1 Pharmacognosy Division, CSIR-National Botanical Research Institute, Rana Pratap Marg, Lucknow, U.P. 226001 India; 2 Botany Department, North Eastern Hill University, Shillong, 793022 Meghalaya India

**Keywords:** Cell division, Cell growth, Cell biology, Computational biology and bioinformatics, Plant sciences

## Abstract

Gloriosine, the predominant metabolite of *Gloriosa superba* L., shares chemical properties with colchicine. We analyze the microtubule-binding affinity of gloriosine at the colchicine binding site (CBS) using an in silico-in vivo approach. The In silico docking of gloriosine showed a binding score of (−) 7.5 kcal/Mol towards β-tubulin at CBS and was validated by overlapping the coupling pose of the docked ligand with co-crystallized colchicine. 2D plots (Ligplot +) showed > 85% overlap between gloriosine and colchicine. The ADMET profile of gloriosine was in accordance with Lipinski’s rule of five. Gloriosine belongs to class II toxicity with anLD_50_ value of 6 mg/kg. In vivo and transmission electron microscopy studies revealed that gloriosine induces abnormalities in cell division such as condensed chromosomes in C-metaphase and enlarged nucleus with increased nuclear material. Gloriosine treated cells exhibited mitotic index of about 14% compared to control of 24% and high anti-proliferative activity i.e. 63.94% cell viability at a low concentration (0.0004 mg/ml). We conclude that gloriosine has a strong affinity for β-tubulin at CBS and thus can be used as a colchicine alternative in cytology and other clinical conditions.

## Introduction

Microtubules are integral parts of the eukaryotic cytoskeleton and are involved in vital functions such as cell motility, cell division and intracellular transport^1^. These are “dynamic polymers” consisting of α and β tubulin, constantly altering between the growth phases “rescue” and shrinkage phase “catastrophe”^1^. During mitosis, the rate of microtubule transition between the two phases increases upto several folds and this dynamic state serves as a sensitive target for microtubule binding agents (MTAs)^1^. Most of these agents have an affinity for β-tubulin and induce abnormal destabilization/ stabilization of tubules leading to mitotic arrest and cell death. MTAs are one of the most important lines of targets for anti-cancer drugs, and there has been intensive research during the past 50 years for discovering small molecules having binding affinity with microtubules^1^. For example, colchicine binding site inhibitors (CBSIs) have been the focus of research for the development of new drugs against cancers^1^. The clinical applications of MTAs as an anti-angiogenic and anti-vascular agent are well established.

Colchicine, a phenethyl iso-quinoline alkaloid is a potent mitotic inhibitor. It is an MTA having an affinity for tubulin monomer and binds at the intermediate functional domain of microtubule assembly, leading to cell arrest during the G_2_/M phase. Animal microtubules are more sensitive towards colchicine compared to plant microtubules, and therefore, very less concentration may be clinically useful to exhibit cytotoxic and immuno-suppressant action^2^. The use of colchicine to induce polyploidy is also well established. It has traditionally been considered as a poison and has an associated risk of toxicity in high doses. However, the stigma of colchicine toxicity came to an end in the year 2009, when USFDA approved it as a drug against gout^3,4^. This has revived the interest in colchicine research and more pharmacological potentials are now being explored^5^. Recently, colchicine was also tried as a potential compound for treating COVID-19 due to its anti-inflammatory activity^6–8^.

Many members of Liliaceae family produce colchicine naturally and *Colchicum autumnale* L. has been its main source to meet the industrial demand. Later, colchicine was isolated from *Gloriosa superba* L., which became its additional commercial source for pharmaceutical industry. The tuber of *G. superba* contains 0.7 to 0.9% colchicine compared to 0.9 to 1.5% in *C.autumnale.* The other important compounds of *G. superba* are colchicosides, superbine, gloriosine, lumicolchicine, 3-demethyl-N-deformyl-N-deacetyl colchicine, 3-demethylcolchicine and N-formyl-deacetyl colchicine^9^. The pharmacological properties of most of the abovementioned metabolites of *G. Superba* other than colchicine are yet to be explored. In the present study, we investigated whether N-deacetyl-N-formyl colchicine (gloriosine) present in *G. superba* having structural similarity to colchicine can serve as a potent mitotic inhibitor. We isolated gloriosine from *G. superba* elite germplasm and studied its affinity towards β-tubulin at the colchicine binding site of colchicine through in silico modeling. Further, the anti-mitotic activity of gloriosine was validated through in vivo assays and transmission electron microscopy (TEM). The ADMET (absorption, distribution, metabolism, excretion, and toxicity) profile of gloriosine was also established. The study scientifically validated a plant-based MTA, which can serve as an alternative to colchicine in cytology and clinical indications.

## Results and discussion

Kumar reported a new alkaloid isolated from *G. superba* that induced chromosome doubling and was provisionally named "gloriosine"^10^. Later, the research trend on *G. superba* and its metabolite was diverted and restricted to colchicine only. The results of in silico docking studies suggest that gloriosine is a promising candidate as a microtubule-binding agent and the activity was validated through in vivo models.

### In silico docking studies

**Figure 1:**
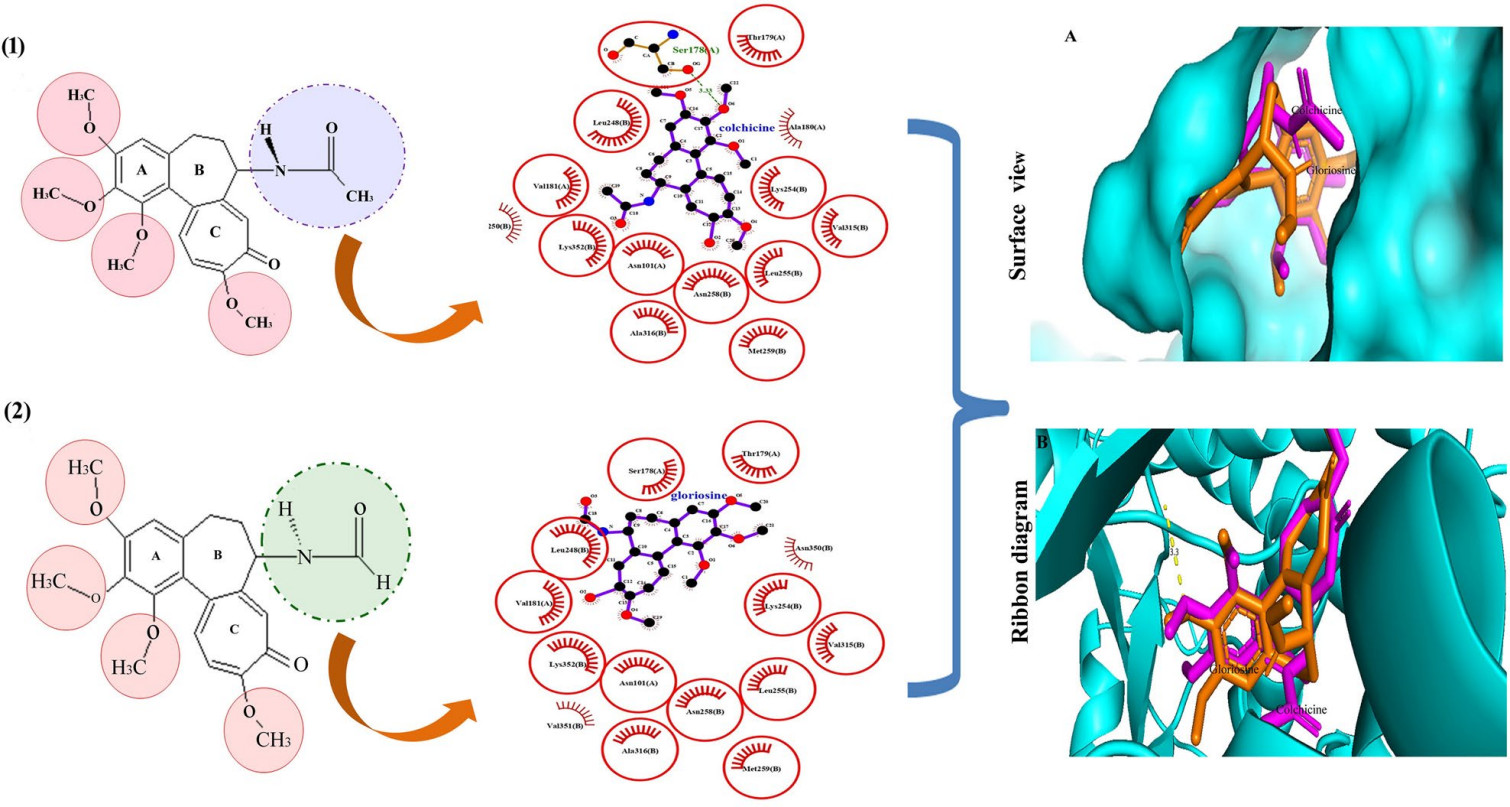
In silico interactions of colchicine and gloriosine at CBS. The chemical structure of colchicine (**1**) and gloriosine (**2**). The Interactions of docked ligands (2D plot) of colchicine (**1**), and gloriosine (**2**) with tubulin protein (1sa0), generated by Ligplot +. Overlapped docking of gloriosine with co-crystallized colchicine in CBS of β-tubulin protein (1sa0). The overlapping was represented from two different sides, i.e. surface view (**A**) and ribbon view (**B**), wherein, gloriosine and colchicine is represented by orange and magenta colour respectively. The enlarged Ligplot + image of colchicine and gloriosine is shown in Supplementary Fig. 1A,B respectively.

Colchicine destabilizes tubulin polymerization during the cell cycle and binds with the β-tubulin at its interface with α-tubulin. This binding leads to distortion in the conformation of αβ-hetero-dimer of tubulin, leading to loss of lateral contacts of the heterodimer, necessary for polymerization, and ultimately leads to tubulin destabilization. Tubulin-Colchicine binding has been extensively studied, and the 'Colchicine Binding Site' (CBS) is well explained^11^. Various phytomolecules of plant origin such as podophyllotoxin, vincristine, and combretastatin have affinity for CBS^12^, and are clinically used as anti-mitotic agents. In the present study, gloriosine showed affinity towards β-tubulin at the CBS. Binding was validated by the overlapping of the docked ligand binding position with co-crystallized colchicine (Fig. 1). Gloriosine is structurally similar to colchicine and consists of a 3, 4, 5-tri methoxy phenyl (ring A), a seven-carbon ring (ring B), and a tropolone ring (ring C). The only difference (in the structures) is the presence of an acetamide group on C-7 of ring B in colchicine and alternatively a formamide group at the same position in gloriosine (Fig. 1). Gloriosine showed a binding score of − 7.5 kcal/Mol, higher in magnitude but statistically insignificant (p = 0.67 at 5% level of significance) than colchicine (− 7.4 kcal/Mol). 2D plots of the docking interactions (of both colchicine and gloriosine) on Ligplot + showed more than 85% overlapping (Fig. 1). Colchicine exhibited hydrophobic interactions with αAsn101, αVal181, αSer178, αThr179, αAla180, βLeu248, βAla250, βLys254, βLeu255, βAsn258, βMet259, βVal315, βAla316, and βLys352. In addition, colchicine also formed a hydrogen bond with αSer178 (Supplementary Fig. S1A). While, gloriosine exhibited hydrophobic (13) interactions with αAsn101, αVal181, αSer178, αThr179, βLeu248, βLys254, βleu255, βAsn258, βMet259, βVal315, βAla316, βAsn350, βVal351 and βLys352 (Supplementary Fig. S1B). It was found gloriosine has the same hydrophobic interaction sites as colchicine, with two additional sites (i.e.; βAsn350, βVal35) and no hydrogen bond. The ring A of colchicine is involved in anchoring with tubulin, while inhibition is modulated by the interactions between ring C and the colchicine binding site^13^. The structural proximity of gloriosine with tubulin indicates a similar binding pattern as that with colchicine. However, it was observed that docking of the gloriosine molecule yields a pose slightly different from colchicine, leading to different 2D plots. This may be either due to the absence of hydrogen bonding interaction between the methoxy group of ring A and tubulin (Fig. 1) or due to the absence of a methyl group in gloriosine at C-7 in ring B. The H-bonding is considered stronger than the hydrophobic interactions, although in the case of gloriosine the better binding score pointed towards the existence of other interactions that needs to be studied. In a previous study, similar results were obtained wherein gloriosine exhibited better binding score than colchicine at the CBS^14^, however the effect was insignificant^14^ and this is in synergy with present findings. The magnitude of binding score varies due to difference in docking programs used for in silico studies. The programs based on two different algorithms^15^ may lead to slight variation in study results. Colchicine has a unique way of interacting with tubulin by involving the α-T5 loop and identification of such interactions in other colchicine derivatives can be path-breaking. We observed that, gloriosine was interacting with the α-T5 loop (comprising of residues 178–180 of the α-tubulin) in a similar way as colchicine. Identification and characterization of such interactions in other colchicine derivatives can be path-breaking^16^. The role of α-T5 loop of colchicine and its derivatives is associated with the better binding energies of their interaction and stabilization with tubulin heterodimer^16,17^. Tubulin isotype βIII was identified as a selective site for screening of drug candidates having anti-tumour activity^18^ and several colchicine derivatives were evaluated in silico^16^.

### ADMET profile of gloriosine

The ADMET (SwissADME software) profile of gloriosine was evaluated to record various parameters such as physicochemical properties, pharmacokinetics, drug-likeness, solubility, and compared those with colchicine. The drug-likeness of gloriosine was found in accordance with Lipinski’s rule of five, wherein the molecular weight is less than 500 Dalton, hydrogen bond donor(s) is not more than 5, hydrogen bond acceptor(s)is less than 10, and consensus logP (an indicator of lipophilicity) is < 5. Gloriosine had good predicted aqueous solubility, high gastrointestinal absorption, did not cross the blood–brain barrier, and the bioavailability score was 0.55 (Table 1). The ADMET profile was also found similar to colchicine (Table 1). The bioavailability of the targeted compound in the Swiss ADME radar (Supplementary Fig. S2) shows that the gloriosine lies in the pink area, which indicates its good bioavailability as a drug candidate. Toxicity studies revealed that gloriosine belongs to the same class as colchicine, i.e., class II with a predicted LD_50_ value of 6 mg/kg (Class II toxic substances are defined as fatal if swallowed, 5 < LD_50_ ≤ 50)^19^. Although both the compounds were toxic against various targets (Table 2), gloriosine was relatively less toxic than colchicine (Supplementary Fig. S2). In silico studies inferred that the docking score of gloriosine was at par with colchicine, having similar binding affinity for β-tubulin at CBS. The overlapped binding pattern of gloriosine with co-crystallized colchicine at the CBS favors that it binds with tubulin in similar fashion as colchicine. The ADMET profile of gloriosine shows high absorption and water solubility, following the Lipinski’s rule of five.

**Table 1:** ADME studies of colchicine and gloriosine generated by SwissADME tool.

S. no	ADME parameters		Colchicine	Gloriosine
1	Phytochemical parameters (Lipinski rule of five)	Mol. Wt	399.44 g	385.41 g
		Hydrogen bond donors	1	1
		Hydrogen bond acceptors	6	6
		No. of rotatable bonds	6	6
		Consensus log P	2.36	2.18
2	Water solubility	Log S (ESOL)	Soluble	Soluble
		Log S (Ali)	Soluble	Soluble
		Log S (SILICOS-IT)	Poorly soluble	Poorly soluble
3	Pharmacokinetics	GI absorption	High	High
		Bioavailability	0.55	0.55
		CYP enzymes inhibitors	Inhibits only CYP2D6 and CYP3A4	Inhibits only CYP2D6 and CYP3A4

**Table 2:** Comparative probability of the toxicity of colchicine and gloriosine on different targets generated by Protox II.

S. no	Toxicity target	Probability of compound toxicity	
		Colchicine	Gloriosine
1	Immunotoxicity	0.99	0.99
2	Cytotoxicity	0.88	0.82
3	Mitochondrial Membrane Potential (MMP)	1.0	0.91
4	ATPase family AAA domain-containing protein 5 (ATAD5)	1.0	0.85

### Isolation of gloriosine from elite germplasm of *G. superba*

**Figure 2:**
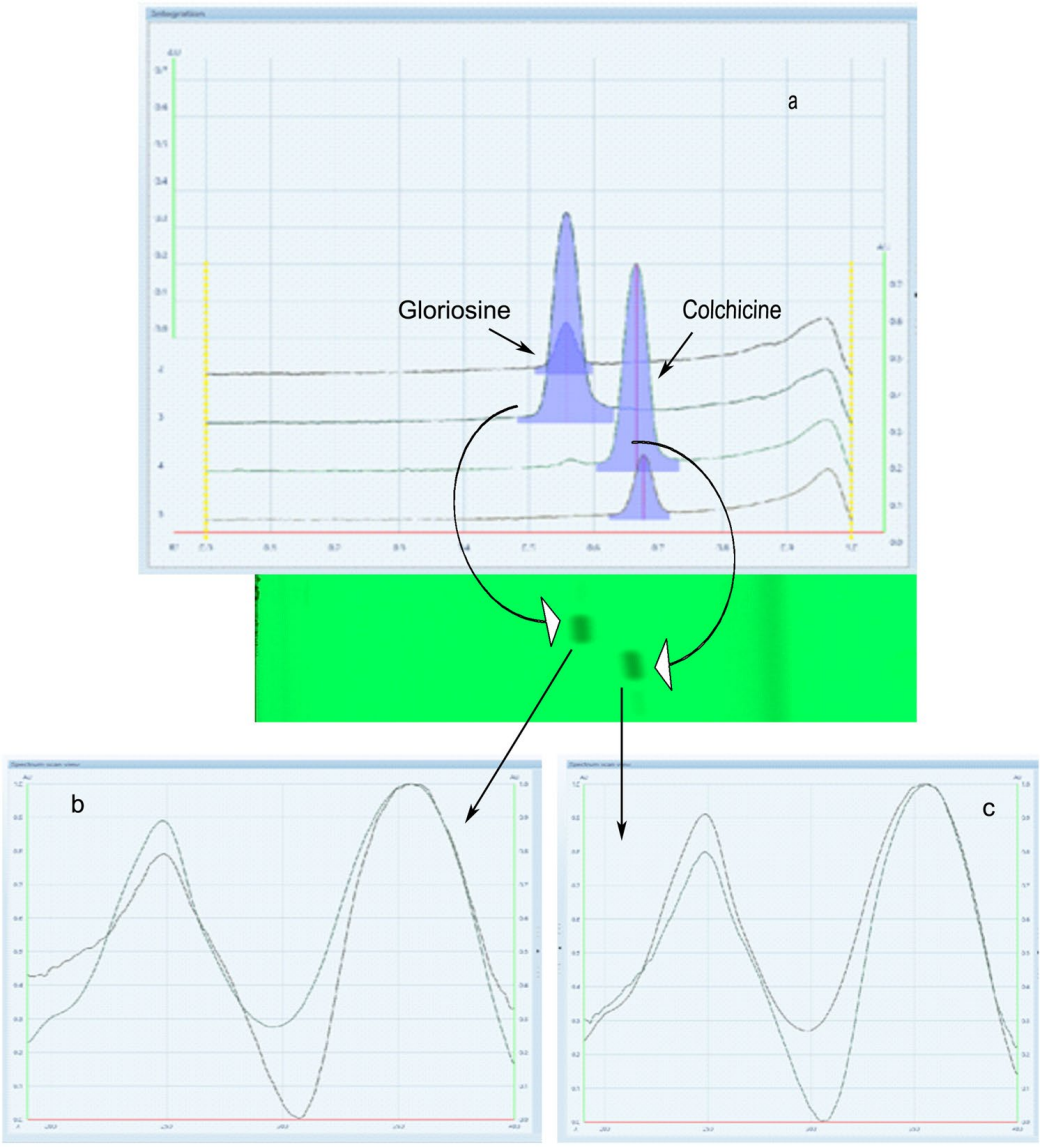
HPTLC quantification of targeted metabolites. The colchicine and gloriosine markers were isolated through preparative TLC and purity of isolated colchicine (Iso1) and gloriosine (Iso2) were estimated using validated HPTLC method. (**a**) Overlay spectra and chromatogram of standard colchicine and gloriosine with Iso 1 and Iso 2. (**b**) Comparison of absorption spectra of reference gloriosine and Iso 2 and (**c**) Absorption spectra of reference colchicine and Iso 1 in the UV–Vis range of 200 to 800 nm.

**Figure 3:**
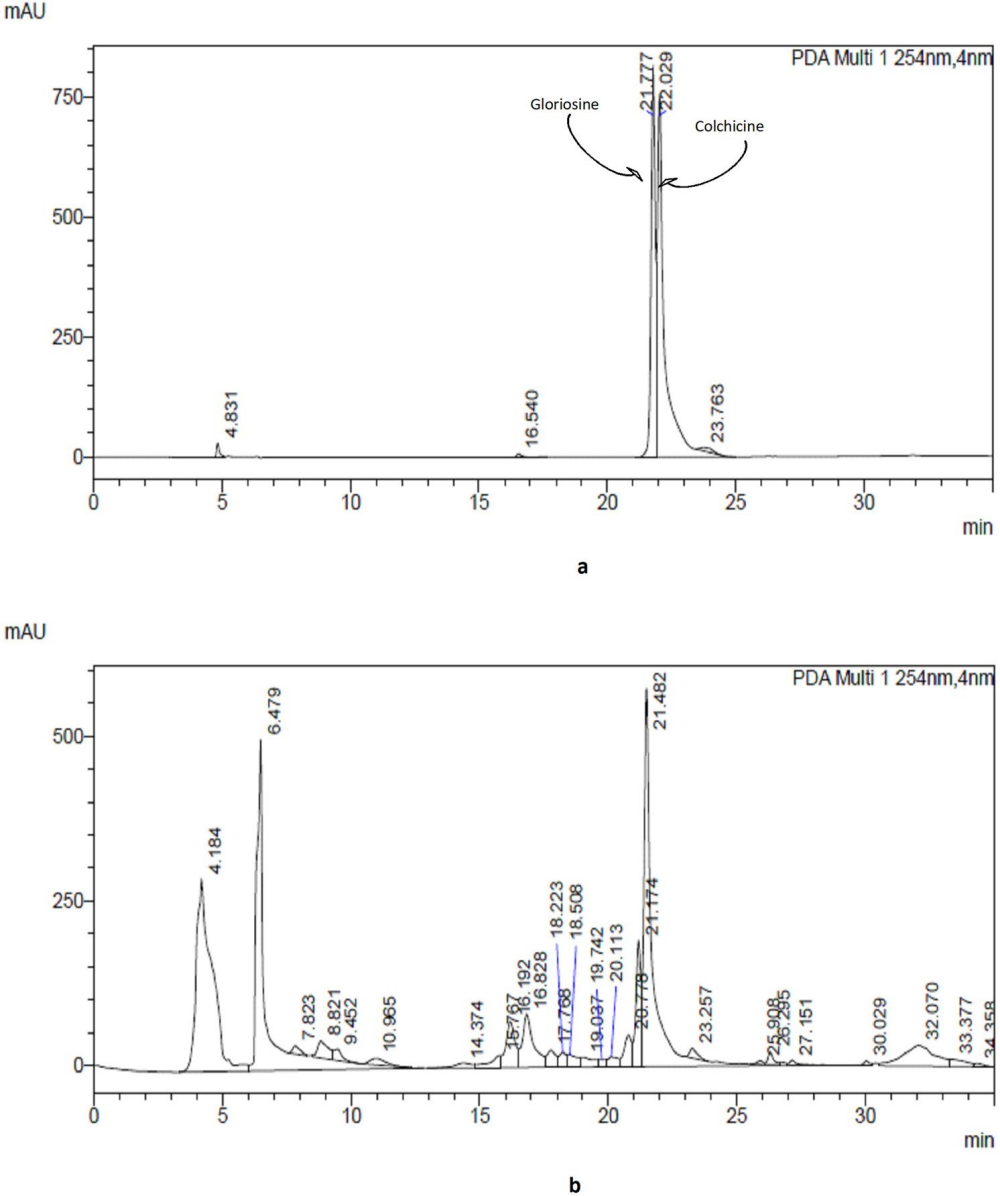
RP-HPLC chromatogram of colchicine and gloriosine. HPLC chromatogram of the standard colchicine and gloriosine (**a**). Identification of colchicine and gloriosine through RP-HPLC in *Gloriosa superba* extract (**b**).

Secondary metabolites produced in plants have innate medicinal properties due to their origin from unique and intricate biosynthetic pathways. We isolated gloriosine from the identified elite germplasm of *G.superba,* and analyzed in vivo anti-mitotic and anti-proliferative activities, and compared those with colchicine. Our group previously recorded intra-specific metabolic variation in *G. superba* collected from various phytogeographical zones of India^20–26^ and elite chemotypes based on gloriosine content were identified^20^. Metabolite profiling of *G. superba* populations across India shows that gloriosine is invariably present in abundance, along with colchicine. A fixed quantity (60 mg) of methanolic extract was charged on PTLC plates and the chromatogram (multiple times) was allowed to develop under the developed protocol^20^. Isolated colchicine (Iso1) and gloriosine (Iso2) were separated at R_f_ 0.66 and 0.55, and the purity of isolated compounds was ~ 97% (Fig. 2). The presence of colchicine and gloriosine in extract (used for isolation) was confirmed through RP-HPLC and separation was performed on a 250 × 4.6 mm (i.d.), 5 µm C18 (Luna phenomenex) column. A 10 mM NaH_2_PO_4_ (pH 3) and acetonitrile as solvent A and solvent B in a gradient ratio (0 min (18% B)), 0–15 min (45% B), 15–25 min (70% B), 25–30 min (25% B) and 30–35 min (25% B) was used at a flow rate of 1 ml/min. The chromatogram ensures the presence of single markers i.e. gloriosine and colchicine at 21.77 and 22.02 min, respectively (Fig. 3).

### In vivo anti-mitotic activity

**Figure 4:**
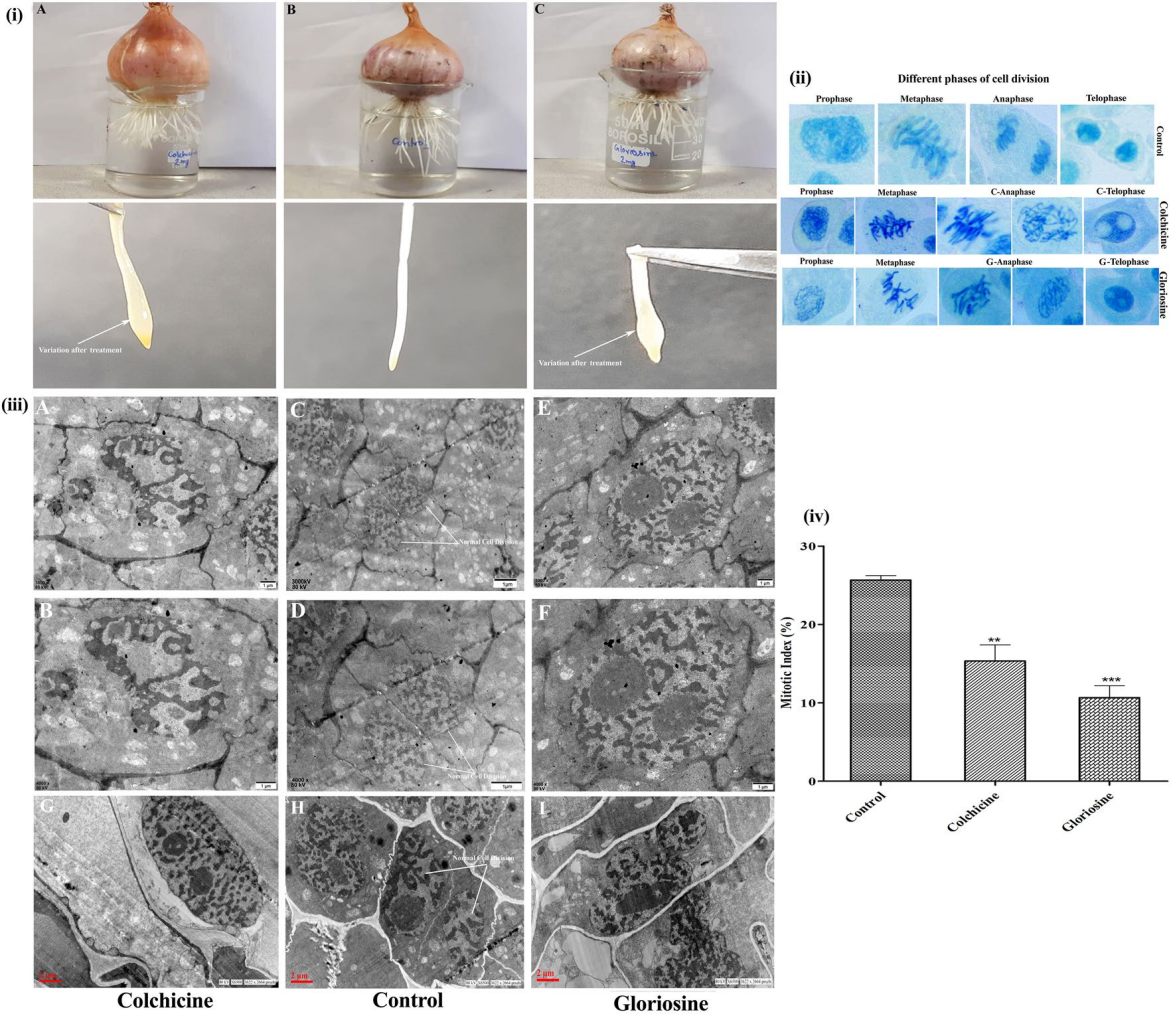
In vivo anti-mitotic and anti-proliferative activity of Iso1 and Iso2. (**i**) *Allium cepa* root tip growing in Iso1 (**iA**), control (**iB**) and Iso2 (**iC**). (**ii**) Different phases of cell division and anomalies induced by three treatments in onion root tip cell. (**iii**) TEM micrographs representing abnormal cell division in Iso1 treated cells (**iii A,B,G**), control (**iiiC,D,H**) and Iso2 treated (**iiiE,F,I**) cells. (**iv**) Mitotic index (%) of treatment after 24 h at 0.05 mg/ml of colchicine (Iso1) and gloriosine (Iso2) was represented by bars and found significant than control. Two and three asterisk denotes significance of treatment from control at p value of 0.0005 and 0.0001 respectively.

Cell division is an inherited process of eukaryotic cells^27^ and microtubule arrangements, which form the cytoskeleton, and are vital for cell functions and viability. The chromosome doubling property of colchicine to obtain polyploidy was first recorded in *Datura stramonium*^28^ and thereafter, it was widely used to induce polyploidy in animal and plant cells. The mechanism of this action is well established^29^ and extensively studied^17,30^. In the present study, in vivo anti-mitotic activity of Iso2 was evaluated in onion root cells and compared with Iso1. Three treatments, namely control, Iso 1, and Iso 2 were applied (Fig. 4). The onion tips treated with control neither show any aberration on external (surface of the onion root tip) morphology, nor any chromosomal abnormality was recorded (Fig. 4) irrespective of the duration of treatment i.e. up to 72 h. Further, microscopic examination of cells revealed that in control treatment, the meristematic zone of the root showed normal mitotic division. The cells on onion root tips were actively involved in cell division and various stages (of the cell cycle) were visible (Fig. 4). Although, the majority of the dividing cells were in prophase (24 ± 2) followed by metaphase (9 ± 1), anaphase (7 ± 1) and telophase (5 ± 1) stages of the cell cycle (Supplementary Table S1). In Iso1 and Iso2 treatments, we observed yellow coloration of onion roots and bulging at the tips, although the lining of tips was intact. Microscopic examination of the groups treated with Iso1 and Iso2 showed that the pattern of cell division changed considerably. The cell cycle arrest was dependent on the concentration and duration of the treatments. Onion cells treated with 0.01 mg/ml of Iso 2 did not show any significant change in chromosome disjunction or mitotic inhibition up to 24 h. However, with an increase in concentration (0.02 to 0.05 mg/ml) and the duration of treatment, i.e., at 48 and 72 h, two type of abnormalities were visible during cell division. Firstly, some cells had initiated division, but uneven chromosome pulling (mitotic inhibition) was recorded along with scattered and condensed chromosomes in metaphase (C-metaphase). It is noteworthy that after Iso2 treatment, most cells were in this condition. Secondly, the cells were undivided with abnormal (due to the effect of treatments) internal morphology i.e. enlarged nucleus with increased nuclear material. This anomaly advocates that gloriosine induces polyploidy. This was seen prominently (Fig. 4) in cells with doubled numbers of chromosomes (in C-metaphase). Further, we observed that the majority of cells were arrested in metaphase and there was a scarcity in the number of cells in the anaphase and telophase stages (Tables 3, 4). We also recorded that with further increase in concentration (i.e. > 0.05 mg/ml) and duration of Iso2 treatment (> 72 h), no significant effect was produced. The pattern of polyploidy induced by Iso2 was similar to Iso1 (Fig. 4).

**Table 3:** Anti-mitotic activity of isolated colchicine (Iso 1) from elite germplasm of *Gloriosa superba*.

Colchicine treatment*	Duration of treatment (h)														
	24					48					72				
Cells stage	P	M	A	T	MI	P	M	A	T	MI	P	M	A	T	MI
0.01 mg/ml	19 ± 2	6 ± 1	8 ± 1	2 ± 0	-	14 ± 1	5 ± 1	4 ± 1	3 ± 0	2 ± 0	13 ± 1	4 ± 1	6 ± 1	3 ± 0	3 ± 1
0.02 mg/ml	15 ± 1	6 ± 1	4 ± 1	1 ± 0	2 ± 0	9 ± 2	4 ± 1	4 ± 1	3 ± 0	5 ± 1	11 ± 1	5 ± 1	4 ± 1	2 ± 0	7 ± 1
0.03 mg/ml	10 ± 2	5 ± 1	4 ± 1	2 ± 0	3 ± 0	10 ± 2	5 ± 1	5 ± 1	2 ± 0	6 ± 1	8 ± 2	6 ± 1	2 ± 0	1 ± 0	6 ± 1
0.04 mg/ml	14 ± 2	5 ± 1	5 ± 1	3 ± 0	3 ± 0	10 ± 1	6 ± 1	4 ± 1	3 ± 0	4 ± 1	7 ± 1	3 ± 1	3 ± 1	3 ± 0	8 ± 1
0.05 mg/ml	13 ± 2	6 ± 1	5 ± 1	1 ± 0	5 ± 1	9 ± 1	4 ± 1	3 ± 0	1 ± 0	6 ± 1	8 ± 1	3 ± 1	2 ± 1	1 ± 0	9 ± 2

*P* prophase, *M* metaphase, *A* anaphase, *T* telophase, *MI* mitotic inhibition. The mean value of number of cells was converted to whole number in case the mean value is in fraction.

*Values are mean (n = 3) ± S.D.

**Table 4:** Anti-mitotic activity of isolated gloriosine (Iso 2) from elite germplasm of *Gloriosa superba*.

Gloriosine treatment*	Duration of treatment (h)														
	24					48					72				
Cells stage	P	M	A	T	MI	P	M	A	T	MI	P	M	A	T	MI
0.01 mg/ml	16 ± 2	5 ± 1	4 ± 1	3 ± 0	2 ± 0	13 ± 1	3 ± 0	2 ± 0	3 ± 1	4 ± 0	9 ± 1	3 ± 1	4 ± 1	1 ± 0	4 ± 1
0.02 mg/ml	14 ± 1	4 ± 1	5 ± 1	2 ± 0	4 ± 0	11 ± 2	3 ± 1	3 ± 1	2 ± 0	6 ± 1	10 ± 1	4 ± 1	4 ± 1	3 ± 0	9 ± 1
0.03 mg/ml	12 ± 2	5 ± 1	3 ± 1	2 ± 0	7 ± 1	11 ± 2	3 ± 1	2 ± 0	3 ± 0	9 ± 2	10 ± 2	2 ± 1	3 ± 0	1 ± 0	11 ± 2
0.04 mg/ml	10 ± 2	6 ± 1	3 ± 1	3 ± 1	8 ± 2	9 ± 1	4 ± 1	2 ± 0	2 ± 0	9 ± 1	9 ± 1	2 ± 1	2 ± 1	1 ± 0	12 ± 1
0.05 mg/ml	11 ± 2	4 ± 1	3 ± 1	2 ± 0	8 ± 1	9 ± 2	3 ± 1	3 ± 0	1 ± 0	11 ± 1	9 ± 1	3 ± 1	2 ± 1	2 ± 0	14 ± 2

Values are mean (n = 3) ± S.D.

*P* prophase, *M* metaphase, *A* anaphase, *T* telophase, *MI* mitotic inhibition.

The effect of Iso2 on cell division was further elaborated by transmission electron micrography and compared with Iso1. In control treated cells, normal eukaryotic cell division was seen. The ultra-micrographs (Fig. 4) of Iso2 treated cells depicted significant variation at the cellular level as compared to control (Fig. 4) and similar to Iso1. In control, the cells were divided normally and different stages of mitotic division were visible with normal nuclei (Fig. 4). In Iso2 treatment, the majority of cells were found arrested during mitotic division, having an enlarged fused nucleus. Due to this, cells were not able to divide into two younger ones and this resulted in polyploidy (Fig. 4). It was previously reported that colchicine affects only the spindle apparatus^31,32^, however the doubling of chromosomes continues but its separation and thereafter movement towards the poles do not take place. And, due to the innate characteristic of a eukaryotic cell, the rest of the phases of cell division continue (undisturbed), resulting in a large nucleus with several micronuclei. In our study, we had a similar observation in gloriosine treated cells (Fig. 4), two micronuclei were seen inhabiting one large nucleus. In colchicine-treated *Allium cepa* cells, numerous binucleate cells were observed with two fused nuclei leading to a tetraploid nucleus^32^. Similarly, binucleate cells and cells with bridged nuclei were also recorded due to the cessation of anaphase movement and the return to interphase of the nuclei without cytokinesis, by colchicine treatment^33^.

The anti-mitotic activity assayed through *the Allium cepa* root tip model is the most accepted in vivo assay due to its resemblance with cell division in normalized and cancer-infected human cells. The indicator used to predict the anti-mitotic activity of Iso2 is the percent mitotic index (Fig. 4, Table 5), which represents the cell proliferation (Table 6) after treatment. In the control group, cells were actively dividing into various phases of mitosis i.e., prophase, metaphase, anaphase and telophase and the mitotic index after 24 h of treatment was 24% ± 3.46. The Iso1 and Iso2 exhibit promising anti-mitotic activity which is evident by the decrease in mitotic index (after 24 h of treatment) in a dose-dependent manner. The lower is the percent mitotic index (MI) at a fixed concentration, the higher is the potency of treatment. The activity potential (MI %) of Iso1 and Iso2 was significantly (α = 0.0001, 0.0005) different from control (Fig. 4). The effect of Iso2 was found at par with Iso1, the magnitude of mitotic inhibition at higher concentration (of Iso2 was found better than Iso1. However, statistically insignificant difference (p = 0.316) at 5% level of significance was recorded among both metabolites at various tested concentrations. Anti-proliferative activity of Iso1 and Iso2 determined against yeast cells showed dose-dependent cell death (Table 6). The cells which remains live after the treatment were found transparent, whereas the dead cells were stained blue (Supplementary Fig. S3). In both the treatments, cell proliferation was reduced to less than 50% as compared to the control. This suggests that Iso1 and Iso2 were not only responsible for cell death but also inhibited cell proliferation. Iso2 was found to have more potential effect, exhibiting high anti-proliferative activity (63.94% cell viability) at low dose i.e. 0.0004 mg/ml. The cell viability in (both) treatments were significantly (p < 0.05) reduced as compared to the control (Supplementary Fig. S4, Table 6), the effect of Iso2 was more pronounced than Iso1, at higher concentrations. The anti-proliferative activity of gloriosine was previously reported on various cancerous and normal cell lines. It exhibit significant cytotoxicity on cancerous cells than normal cells, indicating that gloriosine is less toxic than colchicine. Moreover, in some cell lines, gloriosine was found more active than latter^14^. The molecular mechanism behind the anti-mitotic activity of gloriosine needs to be investigated further in vivo in animal and human subjects for its clinical and biochemical use as an alternative to colchicine.

**Table 5:** Mitotic index of isolated colchicine (Iso 1) and gloriosine (Iso 2) at 24 h after treatment.

Treatment (mg/ml)	Total no. of cells	Total no. of dividing cells	Mitotic index (%)*
Control	200	48	24 ± 3.46
Iso 1			
0.01	200	37	18.5 ± 3.21
0.02	200	29	14.5 ± 1.6
0.03	200	24	12 ± 2.31
0.04	200	32	16 ± 1.52
0.05	200	32	16 ± 1.52
Iso 2			
0.01	200	32	16 ± 1.00
0.02	200	30	15 ± 2.51
0.03	200	31	15.5 ± 1.60
0.04	200	32	16 ± 2.08
0.05	200	28	14 ± 2.31

*Mean value (n = 3) ± standard error.

**Table 6:** Anti-proliferative activity of Iso 1 and Iso 2 in the yeast cell model.

Treatment concentration (mg/ml)	Average no. of live cells	Average no. of dead cells	Average no. of total cells	No. of live cells/mL	Cell viability (%)	Treatment concentration (mg/ml)	Average no. of live cells	Average no. of dead cells	Average no. of total cells	No. of live cells/mL	Cell viability (%)
Control	1466	0	1466	14.66 × 10^7^	100	Control	1466	0	1466	14.66 × 10^7^	100
Iso 1						Iso 2					
0.0001	619	185	804	61.9 × 10^6^	76.99	0.0001	642	241	883	64.2 × 10^6^	72.70
0.0002	640	227	867	64 × 10^6^	73.81	0.0002	574	270	844	57.4 × 10^6^	68
0.0003	470	217	687	47 × 10^6^	68.41	0.0003	502	249	751	50.2 × 10^6^	66.84
0.0004	634	310	944	63.4 × 10^6^	67.16	0.0004	509	287	796	50.9 × 10^6^	63.94

## Discussion

The chemical structures of colchicine and gloriosine share similarity in ring A and ring C, however, there is a minor variation in substitution at the C7 position in ring B. Bhattacharyya^34,35^ and other workers^9^ investigated the tubulin binding interaction, the role of B ring in binding with tubulin fiber, and thermodynamics of B ring analogues in tubulin interaction. The study has proven that the chemical specificity of colchicine-tubulin interaction is independent of the B ring and is related to the methoxy functional group at ring A and ring C. Ring B analogues with and without substitution at C7 bind to CBS in the same way as colchicine and the activity of the molecule also remains intact. However, it is observed that analogues without substituents or with smaller substituents bind remarkably faster than colchicine. Gloriosine possesses a formamide group as the ring B substituent, which is smaller than the acetamide group of colchicine at the same position. It means that the binding affinity of gloriosine to tubulin is likely to be faster than that of colchicine. The work was extended further and the thermodynamics involved in tubulin-colchicine interaction due to the presence of ring B and substitution at C-7 was elaborated^36^. Data suggest that the B ring plays a crucial role in binding properties i.e. kinetics and thermodynamics of this interaction and maintains the dynamics (conformation) and positioning equilibrium of ring A and ring C during the interaction. In vivo results suggest that the anti-mitotic activity of Iso2 was greater than that of Iso1 and perhaps because the methyl group in acetamide substitution at C7 (of ring B) creates an inductive effect^37^ on carbonyl oxygen. However, in the case of Iso2 such an effect was not produced due to the presence of a hydrogen atom i.e. formamide group, and could have provided more stability to the molecule during the interaction process. Furthermore, the formamide group in Iso2 provides an increased stability to the molecule and this leads to a stronger non-reversible gloriosine-tubulin interaction as compared to colchicine. In colchicine, the interaction is poorly reversible^35^. Although this inductive effect was weak (due to the absence of one methyl group only), and therefore the anti-mitotic activity of Iso2 was slightly higher in magnitude, but not significantly different from colchicine. The role of A, B, and C ring (and ring substitutions) of colchicine in CBS binding is well explained, additionally the relative position of C-10 methoxy group and C-9 ketone also plays crucial role with tubulin binding^38^. Gloriosine possesses structural similarity with colchicine at these two ends also. Therefore, considering the structural similarity with colchicine molecule, in silico docking profile of gloriosine at β-tubulin in CBS revealed that gloriosine had a strong affinity for the colchicine binding site (CBS) and docked(gloriosine-tubulin interaction) well like colchicine. The ADMET profiles of both molecules were quite similar, gloriosine however was less toxic than colchicine. Therefore, we conclude that gloriosine is at par with colchicine and is a potential MTA.

The anti-mitotic activity of gloriosine was validated through *the invivo* model. To evaluate the intrinsic activity of the naturally occurring gloriosine molecule and compare it with colchicine, gloriosine was isolated from the elite germplasm of *G.superba*, selected through an earlier chemotaxonomic study on species populations in various phytogeographical zones of India. The in vivo anti-mitotic activity of isolated gloriosine (Iso2) in onion cells induced chromosomal abnormality and inhibited cell division. Microscopic examination showed that the pattern of cell division changed considerably and the arrest of the cell cycle was found dependent on the concentration and duration of treatment. Abnormal chromosome pulling with scattered and/or condensed chromosomes and undivided cells with enlarged nuclei were recorded. The anti-mitotic index and anti-proliferative activity of Iso2 was at par with Iso1. The pattern of polyploidy induced by Iso2 suggest that the underlying mechanism of action is due to interaction with tubulin, like colchicine.

## Conclusion

In nutshell, *insilico* docking of gloriosine on CBS and further validation through the in vivo assay and TEM indicated that gloriosine has potential anti-mitotic activity like colchicine and, therefore, it can be used as a microtubule-binding agent. The molecule induces polyploidy, however further studies are needed to confirm its efficacy in clinical conditions through in vivo animal/ human studies and for understanding the underlying mechanisms of action.

## Methods

### Chemical and reagents

Standards, viz. colchicine and N-deacetyl-N-formyl colchicine were obtained from M.P. biomedical (California, USA) and Toronto research chemicals (Canada). The other chemical and reagents used for quantification of the metabolites and in vivo assays were purchased from Merck (HPLC and AR grade).

## In silico molecular docking

The anti-mitotic activity of gloriosine was studied through in silico docking against tubulin to analyze its colchicine-like inhibitory effects on tubulin polymerization. The binding efficiency of colchicine and gloriosine to tubulin fiber was analyzed through the Autodockvina program 1.5.7 (http://vina.scripps.edu/)^39,40^. For the3D structures of the ligands, colchicine (PubChem ID 6167) and gloriosine (PubChem ID 23890) were downloaded in SDF (structure data file) format from PubChem databases and were converted to PDB (protein data bank) format using Open Babel tool (http://cheminfo.org/Chemistry/Cheminformatics/FormatConverter/index.html). For tubulin protein structure, tubulin-colchicine stathmin-like domain complex structure (PDB ID ISA0) was downloaded from the RCSB-PDB database (https://www.rcsb.org/). All the co-crystallized ligands, chains C, D and E were removed from the protein and were further optimized along with the ligands using Autodock Tools 1.5.7^40,41^.All the ligands and the protein were converted to the ".pdbqt" format for docking. The grid maps were centered on the colchicine binding site, docking was performed with a grid box of 62 × 62 × 62 points and exhaustiveness of 64. Generated Protein–ligand interactions were analyzed and illustrated using LIGPLOT + v1.4.5^42^ For 3D visualisation, the generated output files were opened in PyMOL (The PyMOL Molecular Graphics System, Version 2.0 Schrödinger, LLC.sx) and interactions between ligand and protein were visualized in their respective binding pockets.

### ADMET studies

Drug likeness studies, pharmacokinetics, bioavailability, and ADME (absorption, distribution, metabolism and excretion) studies were performed using SwissADME^43^. The gloriosine toxicity profile was analyzed and compared with colchicine using ProTox-II^44^.

### Plant material, extraction and Isolation protocol

Fresh tubers of *G. superba* were collected from the natural population (elite germplasm) of the western ghats of India following the Good Field Collection Practices (GFCP) guidelines of the National Medicinal Plant Board, Govt. of India^45^. The samples were shade-dried and extracted with methanol through the cold maceration technique. Accurately weighted (methanol) extract was applied on 20 × 20 cm glass PTLC plates (preparative thin layer chromatography), 0.5 mm silica gel 60 UV_254_ nm (Merck PTLC plates). The sample was applied as one long continuous band, across the plate length (18 cm) and separation was carried out on a tertiary solvent system of chloroform: acetone and diethyl amine (5:4:1 v/v/v) along with standard markers^20^. The developed chromatogram was visualized under UV_254_ and the target markers were identified and scrapped individually from the plate. The adsorbed silica was dissolved in 2–5 ml of methanol and filtered with a 0.45micron (Millipore) filter to separate the silica particles. The filtrate was then applied on PTLC plates and the procedure was repeated until a pure band was observed for each marker *i.e.* isolated colchicine (Iso1) and isolated gloriosine (Iso2). The yield of isolated compounds was quantified, expressed in percentage and peak purity was estimated via HPTLC.

### Anti-mitotic activity

#### Allium cepa root tip model

The anti-mitotic activity was estimated by *the A. cepa* root tip model^46^ with slight modifications. *A. cepa* (50 ± 5 g) were incubated in double distilled water, in glass beakers. The bulbs were allowed for germination in the dark (24 h.) at room temperature (25 °C ± 3), till the roots were grown up to 2 cm long and were then transferred into beakers containing control and variable concentrations of (0.01, 0.02, 0.03, 0.04 and 0.05 mg/ml) Iso1 and Iso2 (5 onions in each group). The incubation was continued for a time interval of 24 h, 48 h and 72 h after treatment, the time of transfer (from water to treatment groups) is considered as zero hr. After the incubation period, 2–3 longest meristematic roots from each treatment were harvested with a sharp razor blade at a distance of 4–7 mm from the meristematic zone of the root and immediately fixed in a fixative, hydrochloric acid: ethanol (1:1) for 2 min. In addition, the tissue was washed with distilled water and then slightly warmed in 0.5% toludine blue solution. The root tips were placed on a microscopic slide and the meristematic zone was slices, extra part was discarded and washed with double distilled water, followed by gentle tapping to make squash and the access water was removed by blotting paper. The squash was then covered with a cover slip and different stages of mitosis were observed under a light microscope (40x). Photo-micrographs were taken with the digital microscope (Nikon, Japan, Model-Eclipse Ci).

#### Determination of mitotic index

To determine the mitotic index, the root tips (2 to 3 mm) were sliced and fixed in a binary solution of acetic acid (45%) and 1 N HCl (9:1). The tips were then softened and stained with 0.1% methylene blue. For the analysis of each tip, 200 cells (approx.) were counted in 5–8 fields under microscopic magnification, (40x) and the cells expressing the various stages of mitosis were counted. The mitotic index was counted using the formula (P + M + A + T)/total cells, where alphabets denote prophase, metaphase, anaphase and telophase respectively.

#### Transmission electron microscopy

Transmission electron microscopy (TEM) observations of treated root tips (apex region) were used to examine the cellular variations/alterations induced by Iso1 and Iso2 (after 24 h of treatment) compared to control (distilled water). Briefly, the apex regions of the root tips (morphologically distinct) were sliced into small segments (2–3 mm) and the sample was prepared as described^47^. Root tips (three biological replicates) were investigated using transmission electron microscopy (JEM1400 Transmission Electron Microscope (JEOL Co., Tokyo, Japan). The experiments were executed twice along with three independent replicates, and one represented micrograph was selected.

### Anti-proliferative assay

The anti-proliferative potential of Iso1 and Iso2 was determined using a yeast cell model^48^ with slight modifications as follows.

#### Yeast inoculum

Approximately 5 g of commercially available yeast was added to 100 ml of sterile nutrient broth in a sterilized conical flask and incubated at 37 °C for 24 h. 1 ml of seeded broth was diluted with distilled water (up to 10 ml) for achieving the cell concentration of 25.4 × 10^4^.

#### Cell viability count

In a test tube, potato dextrose broth (PDB, 2.5 ml) was mixed with extract dilution (1 ml) and yeast inoculum (0.5 ml), while the control contained only PDB and yeast inoculum. All test tubes were incubated for 24 h at 37 °C and after incubation, the cell suspension in each tube was mixed with 0.1% methylene blue and observed under low power (10x) microscope. The number of living cells (which did not have staining and appeared transparent) and dead cells (stained and appeared blue) were counted for the control and the samples were treated with different concentrations of Iso1 and Iso2 in 16 chambers of the hemo-cytometer and the mean was determined. The number of cells/ml and cell viability (%) were calculated.

### Statistical analysis

The data were analysed using Excel-stat (2010) and each observation was taken in triplicate and treatments were compared using Analysis of Variance (ANOVA) at a 5% level of significance.

## Supplementary Information

Supplementary Information.

## Data Availability

All data generated or analysed during this study are included in the article.
