# Supplementary material for: Docking experiments suggest that gloriosine has microtubule-targeting properties similar to colchicine
Source: Sci Rep. 2023 Mar 24;13:4854. doi: 10.1038/s41598-023-31187-6 (PMC10038372; doi:10.1038/s41598-023-31187-6)
Supplement: Supplementary file 1 — Supplementary Information. [file 41598_2023_31187_MOESM1_ESM.pdf]

Docking experiments suggest that gloriosine has microtubule-targeting properties similar to colchicine

Ankita Misra[1], Mridul Kant Chaudhary[1], Satyendra Pratap Singh[1], Deepali Tripathi[1], Saroj Kanta Barik[2] and Sharad Srivastava[1*]

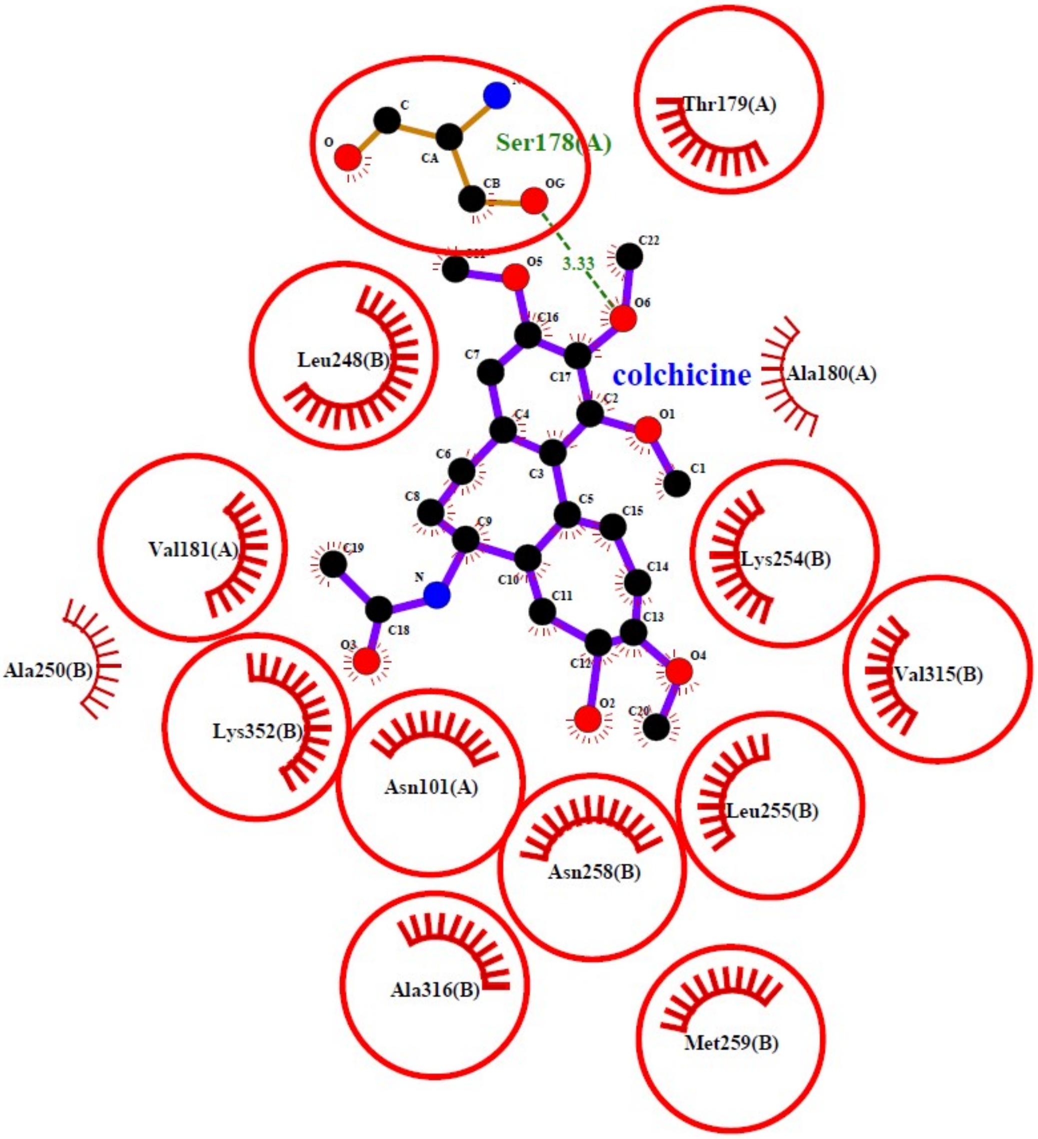

Supplementary Figure S1A. Enlarged view of Ligplot of colchicine-tubulin interaction.

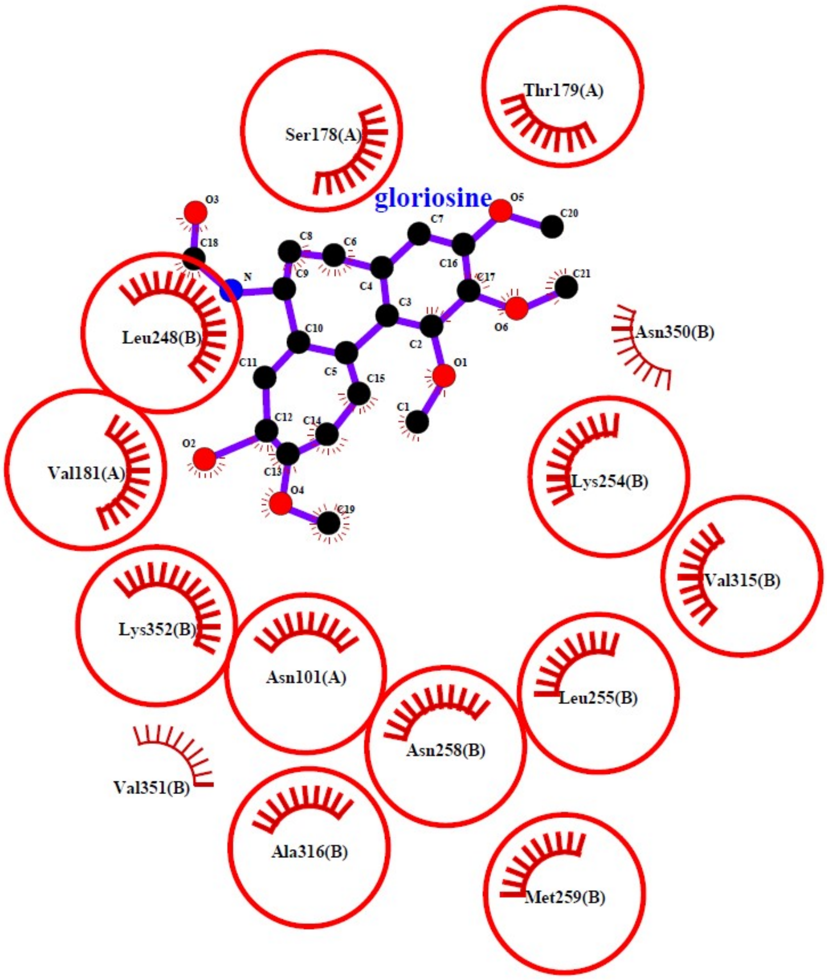

Supplementary Figure S1B. Enlarged view of Ligplot of gloriosine-tubulin interaction.

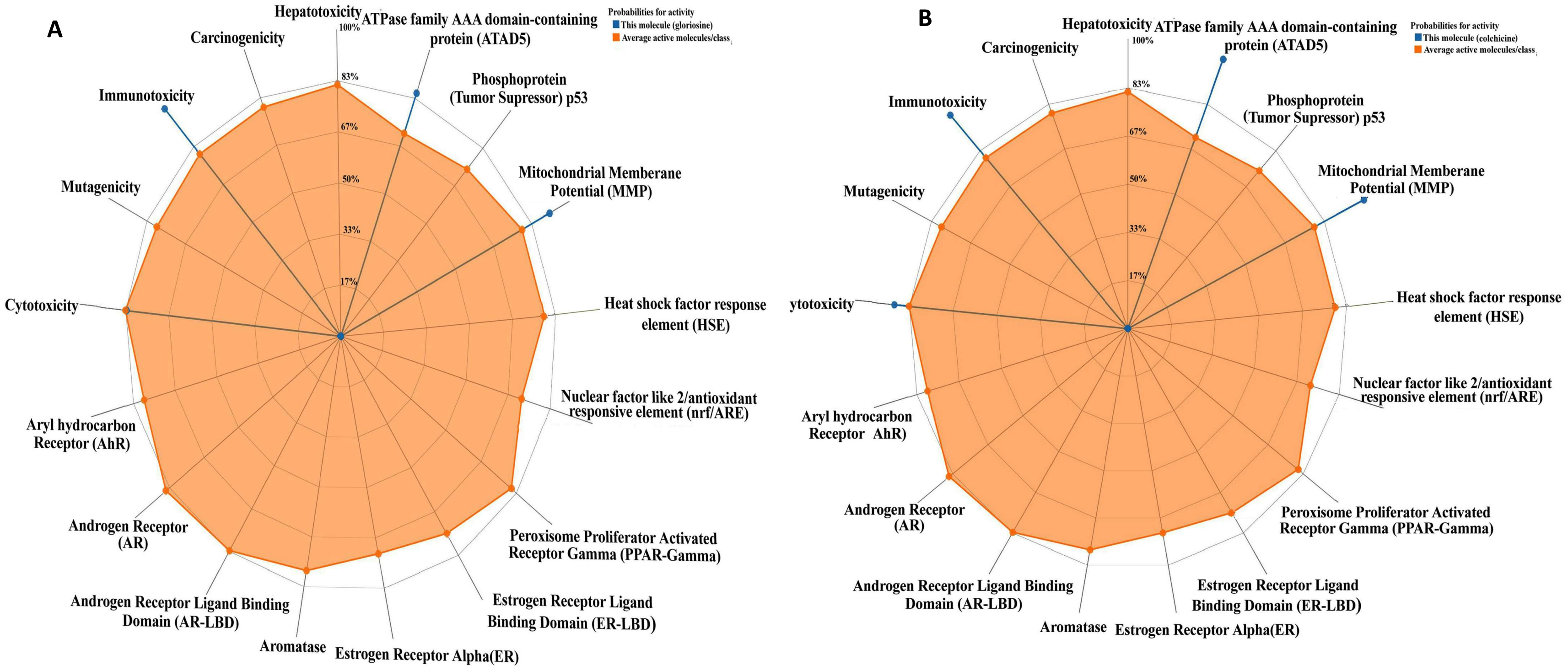

Supplementary Figure S2. Toxicity radar of colchicine and gloriosine generated from ADMET studies (Protox II tool). A. Gloriosine. B. Colchicine.

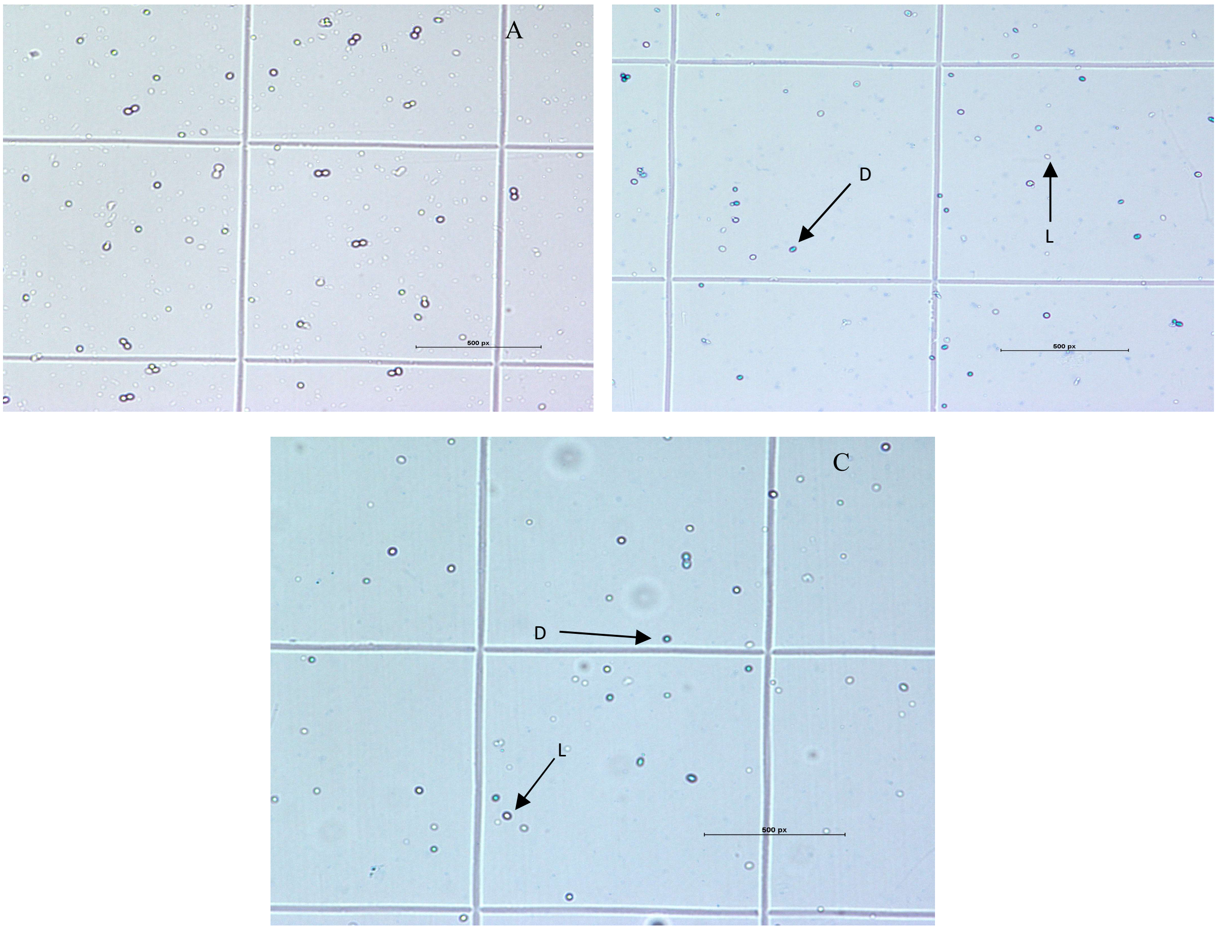

Supplementary Figure S3. Evaluation of anti-proliferative activity of *Saccharomyces cerevisiae* treated with control (A), Iso 1 (B), and Iso 2 (C) at 600 px (pixel). L represents living cells, which are transparent and D represents blue stained dead cells when treated with 0.1% methylene blue. The images are observed under low power (10x) microscope.

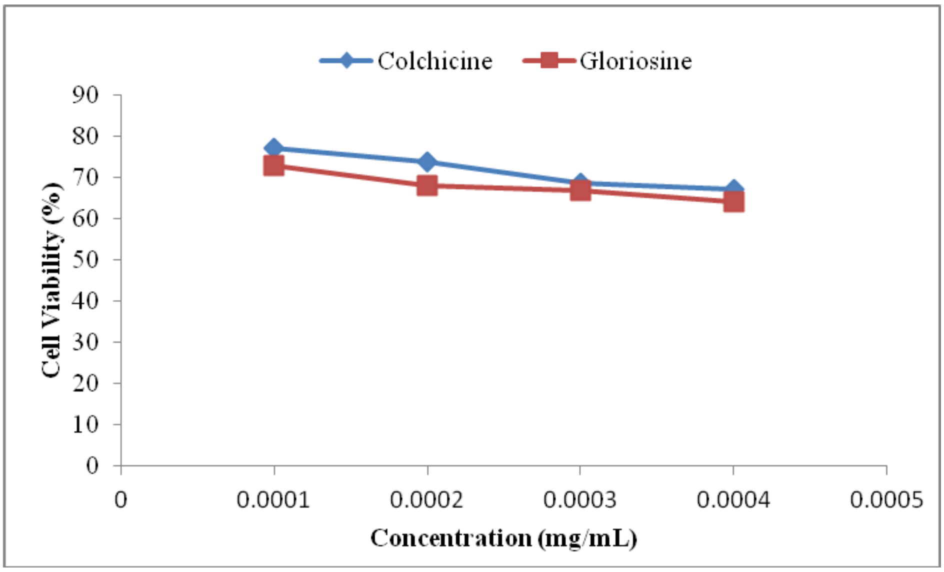

Supplementary Figure S4. Anti-proliferative activity of Iso1 and Iso2 treatment on the cell viability of S*accharomyces cerevisiae* cells. Line graph represents that with increase in concentration of treatment, the cell viability decreases.

Supplementary Table S1. Anti-mitotic activity in control, representing the number of cells in various phases of cell division.

Control*	Prophase	Metaphase	Anaphase	Telophase
**No. of Cells**	24 ± 2	9 ± 1	7 ± 1	5 ± 1

*n=3, the mean value of number of cells was converted to whole number in case the mean value is in fraction.
